# Perinatal programming of neuroendocrine mechanisms connecting feeding behavior and stress

**DOI:** 10.3389/fnins.2013.00109

**Published:** 2013-06-17

**Authors:** Sarah J. Spencer

**Affiliations:** School of Health Sciences and Health Innovations Research Institute, RMIT UniversityMelbourne, VIC, Australia

**Keywords:** hypothalamic-pituitary-adrenal axis, glucocorticoids, development, leptin, insulin

## Abstract

Feeding behavior is closely regulated by neuroendocrine mechanisms that can be influenced by stressful life events. However, the feeding response to stress varies among individuals with some increasing and others decreasing food intake after stress. In addition to the impact of acute lifestyle and genetic backgrounds, the early life environment can have a life-long influence on neuroendocrine mechanisms connecting stress to feeding behavior and may partially explain these opposing feeding responses to stress. In this review I will discuss the perinatal programming of adult hypothalamic stress and feeding circuitry. Specifically I will address how early life (prenatal and postnatal) nutrition, early life stress, and the early life hormonal profile can program the hypothalamic-pituitary-adrenal (HPA) axis, the endocrine arm of the body's response to stress long-term and how these changes can, in turn, influence the hypothalamic circuitry responsible for regulating feeding behavior. Thus, over- or under-feeding and/or stressful events during critical windows of early development can alter glucocorticoid (GC) regulation of the HPA axis, leading to changes in the GC influence on energy storage and changes in GC negative feedback on HPA axis-derived satiety signals such as corticotropin-releasing-hormone. Furthermore, peripheral hormones controlling satiety, such as leptin and insulin are altered by early life events, and can be influenced, in early life and adulthood, by stress. Importantly, these neuroendocrine signals act as trophic factors during development to stimulate connectivity throughout the hypothalamus. The interplay between these neuroendocrine signals, the perinatal environment, and activation of the stress circuitry in adulthood thus strongly influences feeding behavior and may explain why individuals have unique feeding responses to similar stressors.

## Introduction

How an individual responds to stress and how this influences their feeding behavior is governed by many factors, including genetic influence and the proximal environment. For instance, body mass index, an outcome closely associated with diet (Duvigneaud et al., 2007; Wan et al., 2009), is thought to be 40–70% heritable (Loos, 2009), but a person's social group also has significant influence over their food choices (Dabbaghian et al., 2012; Robinson and Higgs, 2012) and there is even seasonal variation in food intake, with people consuming more fat in the winter and spring (Van Staveren et al., 1986; Watson and McDonald, 2007). In addition to these examples, immediate life stress can influence feeding behavior. For example, perceived stress over long periods, such as economic difficulties and job-related demands, is associated with excess weight gain (Block et al., 2009; Fowler-Brown et al., 2009). Social status in humans, and chronic social subordination and reorganization of the social group in macaques, is linked with obesity, increased central (visceral) fat, and indices of metabolic syndrome (Shively and Clarkson, 1988; Jayo et al., 1993; Brunner et al., 1997; Shively, 1998; Shively et al., 2009). These types of stressors encourage negative eating behaviors that are likely to precipitate or contribute to weight gain. Thus, those with high job stress are more likely to eat until they are full and more likely to eat to control mood (Nishitani et al., 2009).

Stress and elevated glucocorticoids (GC) also tend to encourage appetite specifically for high energy highly palatable foods (La Fleur et al., 2004; Warne et al., 2006, 2009; Dallman, 2010). Rats exposed to chronic stress (daily 3 h restraint stress for 5 days) prefer calorically dense foods, such as lard and sucrose, relative to non-stressed rats (Pecoraro et al., 2004). Hypothalamic-pituitary-adrenal (HPA) axis responses to restraint are even attenuated by these high calorie foods, indicating such “comfort eating” can actually help control HPA axis reactivity to stress (Pecoraro et al., 2004). This ability of high energy foods to ameliorate stress responses is reinforced by the influence of foods on reward pathways (Bassareo and Di Chiara, 1997, 1999). Similar brain circuitry is recruited by calorically dense food as by drugs of addiction and, in this regard, the drive to eat highly palatable food is significantly correlated with the drive to consume drugs of abuse (Gosnell, 2000; Nieuwenhuizen and Rutters, 2008; Coccurello et al., 2009). These reward outcomes are largely mediated by the nucleus accumbens. Nucleus accumbens dopaminergic, opioid, and glutamatergic transmission, for instance, are important for mediating reward-associated information, irrespective of whether it is related to drugs (Di Chiara and Imperato, 1988; Koob and Le Moal, 2001; Ito et al., 2004), sex (Balfour et al., 2004), or palatable food (Berridge, 2009). Additionally, the only peptide known to stimulate food intake, ghrelin (Hosoda et al., 2006), promotes the rewarding feeling food gives (Egecioglu et al., 2010) and is also involved in the feeling of reward elicited by alcohol and psychostimulant drugs (Jerlhag et al., 2009, 2010).

Despite direct lifestyle factors influencing feeding, it is also clear that given similar diets, activity levels, and life stress, individuals do not necessarily respond with the same feeding style or maintain the same body weights (Eilat-Adar et al., 2005; Matsuo et al., 2009). It is now evident the perinatal environment is equally, if not more, important in programming how an individual responds to stress, its feeding patterns, and how stress and feeding influence one another. The early life environment may therefore account for some of these inter-personal differences. This review will discuss the impact of the perinatal environment on the interplay between stress and feeding behavior.

## Early life stress programs the hypothalamic-pituitary-adrenal (HPA) axis and hypothalamic feeding circuitry to influence feeding behavior long term

### Prenatal stress

It is now clear that early life stress is an important programming factor in a number of aspects of physiology. Foetal, neonatal, and postnatal stress are certainly important in determining adult vulnerability to dysfunction in the neuroendocrine systems regulating both the HPA axis and food intake under stressed and non-stressed conditions.

During pregnancy the foetus is relatively protected from the effects of stress experienced by the mother. In pregnancy, progesterone and its metabolite, allopregnanolone, are increased in the maternal brain. Allopregnanolone usually inhibits the noradrenergic input to the paraventricular nucleus of the hypothalamus (PVN) from the nucleus of the solitary tract, which stimulates the HPA axis response to stress (systemic interleukin 1β). This inhibition is enhanced with the higher concentrations of allopregnanolone, and the response to stress further inhibited (Brunton et al., 2005, 2009; Brunton and Russell, 2011). Thus, pregnant animals have a smaller increase in corticosterone in response to stress than non-pregnant animals do (Brunton et al., 2005; Slattery and Neumann, 2008; Brunton et al., 2009). In addition, during pregnancy the placenta produces significant amounts of 11β-hydroxysteroid dehydrogenase (HSD)2, which converts GC from the active to the inactive form before they can reach the foetus, protecting it from GC in the maternal circulation (Lucassen et al., 2009).

Such elaborate mechanisms to protect the foetus from the effects of stress would suggest such effects are likely to be dangerous, and certainly in cases of severe or chronic maternal stress excess GC may still negatively affect the foetus. Severe stress (e.g., death of a close relative in humans or restraint in rodents) during pregnancy can affect foetal brain development (Henry et al., 1994; Rossi-George et al., 2009), can influence mood regulation and predispose to anxiety behaviors (Vallee et al., 1997), can disrupt learning and memory (Lordi et al., 1997; Entringer et al., 2009), and can enhance sensitivity to drug abuse (Morley-Fletcher et al., 2004; Thomas et al., 2009).

Prenatal stress can also influence feeding behavior long-term. Babies born to mothers who experienced the death of a family member immediately before or during pregnancy have a significantly increased risk they will gain excess weight in childhood (Li et al., 2010). These prenatal stress effects are also evident in animal models (Tamashiro et al., 2009). Rat pups born to dams that were exposed to chronic variable stress during pregnancy are hyperphagic immediately after birth (Purcell et al., 2011) and throughout life when fed normal rat chow (Pankevich et al., 2009).

These effects of prenatal stress are associated with short- and long-term changes in GC regulation and the HPA axis. In the short term, chronic variable stress reduces 11β-HSD2 levels in the placenta of the female foetus, allowing GC access to influence the foetus (Pankevich et al., 2009). Prenatal restraint stress given to rat dams also leads to dysregulated corticosterone responses to mild stress in the offspring when they are tested in adulthood (Henry et al., 1994; Maccari et al., 1995, 2003; Koehl et al., 1999; Rossi-George et al., 2009). This effect on HPA axis function is likely to be due to reduced mineralocorticoid and glucocorticoid receptor (GR) expression at the level of the hippocampus (Henry et al., 1994; Maccari et al., 1995) and is linked with susceptibility to obesity and indices of diabetes if the animals are later fed a high fat diet (Tamashiro et al., 2009). Similarly, prenatal dexamethasone (a synthetic GC) leads to hyperactivity of the HPA axis and to obesity long-term (Dahlgren et al., 2001).

### Postnatal stress

Postnatal stress can also lead to a predisposition to HPA axis dysfunction, appetite dysregulation, and obesity in adulthood. Early psychological trauma in humans such as sexual abuse, the death of a mother or significant family member, or parental stress, is a significant risk factor for the development of obesity in later life (Koch et al., 2008; D'Argenio et al., 2009). Children from families reporting high levels of stress are significantly more likely to be obese than those from families without such stress (Koch et al., 2008; Moens et al., 2009). Similarly, exposure to war-related events during childhood has been associated with a higher body mass index later in life (Llabre and Hadi, 2009).

In rodents, the postnatal period is also one of particular vulnerability to the programming effects of stress and GC. In the rat, the first to second weeks of life are distinctive as a stress hypo-responsive period (Sapolsky and Meaney, 1986). At this time, the rat has low basal corticosterone and adrenocorticotropic hormone (ACTH) concentrations. It also does not respond to stress to the same degree as adult rats do (Levine, 2002), an effect that is likely due to an extremely efficient GC negative feedback response (Dent et al., 2000; Vazquez et al., 2006). In humans, this period is approximately analogous to the first 12 months of life (Gunnar and Donzella, 2002). The stress hypo-responsive period is contingent upon the continued presence of and feeding from the dam (Tilbrook et al., 2006) and parenting style during this time, categorized by high- or low-intensity nursing and grooming, can predict later-life responses of the HPA axis to stress (Liu et al., 1997; Champagne and Meaney, 2001). The stress hypo-responsive period and HPA axis development can also be affected by early life stress.

In rodents, separation from the mother is a significant psychological stress to the neonate and has long-lasting physiological effects. It leads to anxiety in adulthood, affects basal plasma corticosterone and ACTH, alters GR expression in the brain, and exacerbates the corticosterone response to stress (Lehmann et al., 2002a,b; Barna et al., 2003; Xu et al., 2011). Maternal separation of this type in early life can lead to a life-long reduction in food intake and a significant aversion to foods high in carbohydrates, compared with no maternal separation (Penke et al., 2001). Maternal separation also increases vulnerability to the effects of stress on feeding regulation. So, in a study where maternal separation or social isolation alone were insufficient to affect feeding behavior, food intake was significantly increased, as was weight gain, in socially isolated rats that had previously undergone maternal separation (Ryu et al., 2009).

Perinatal stress thus clearly influences HPA axis function long-term and this can significantly impact upon feeding behavior. However, other early life perturbations can also influence this circuitry. Perinatal nutrition is an important example.

## Early life nutrition programs HPA axis and hypothalamic feeding circuitry to influence feeding behavior long term

### Prenatal nutrition

Before an infant is even exposed to the semi-direct influence of diet or stress from the mother, it inherits a dietary legacy from the father that can contribute to the development of stress- and feeding-related pathways. Obesity in the father, for instance, can damage the sperm; reducing concentration, motility, and morphology, and contributing to DNA damage (Kasturi et al., 2008), damage that can be ameliorated by diet and exercise interventions (Palmer et al., 2012). Paternal obesity increases the risk a baby will be born small (Power et al., 2003) and, in girls, increases the risk of higher adiposity levels pre-puberty (Figueroa-Colon et al., 2000). High fat diet in the sire in rodents can also induce β-cell dysfunction long-term in the offspring contributing to the development of diabetes symptoms (Ng et al., 2010).

Unsurprisingly, maternal diet and adiposity also contribute to the offspring's development (Guillaume et al., 1995; Parsons et al., 2001). There is a clearly established relationship between maternal obesity and offspring obesity (Dabelea et al., 2000; Ruager-Martin et al., 2010) and the children of obese mothers are more likely to develop metabolic complications such as diabetes in later life (Dabelea et al., 2000; Boney et al., 2005). There is also evidence to suggest obesity *per se* is not necessary to influence offspring feeding patterns and metabolism. A maternal diet that is high in fat or a maternal “junk food” diet leads to malformation of central reward pathways in the offspring. The rewarding nature of food is heightened and these offspring come to preferentially select high fat, high sucrose foods (Ong and Muhlhausler, 2011; Gugusheff et al., 2013). This diet in the mother leads to hyperinsulinaemia, insulin resistance, and increased fat deposition in the offspring (Albuquerque et al., 2006; Srinivasan et al., 2006; Ashino et al., 2012).

Severe prenatal malnutrition is a unique type of nutritional stress that can also program changes to the HPA axis and feeding circuitry. The Dutch Famine, or Dutch Hunger Winter, of 1944–1945 was a devastating period of serious malnutrition and starvation as a result of the final battles and aftermath of World War II that affected much of the population of the Netherlands. Studies of victims of this disaster have revealed that inadequate nutrition in the first trimester of gestation (but not the last trimester) leads to significant obesity and metabolic sequelae in young adult males (Ravelli et al., 1976) and middle-aged females (Ravelli et al., 1999; Roseboom et al., 2000a,b). This prenatal under-nutrition also predisposes people to prefer a diet that is high in fat (Lussana et al., 2008). These findings are mirrored by similar results in animal models of intrauterine growth restriction. Thus, intrauterine growth-restricted rodents eat more than controls and have a preference for highly palatable fatty foods (Vickers et al., 2000; Bellinger et al., 2004; Bellinger and Langley-Evans, 2005). For example, rats born to dams fed a low protein diet during pregnancy chose to eat more of a high fat and less of a high carbohydrate diet than control rats did (Bellinger et al., 2004). *In utero* malnutrition can also lead to a reduction in physical activity after birth (Vickers et al., 2003; Sebert et al., 2009), both these factors leading to obesity and comorbidities (Jimenez-Chillaron and Patti, 2007).

It is likely that, as with maternal stress, maternal nutrition regulates placental 11β-HSD2 levels to influence foetal exposure to GC (Stocker et al., 2004, 2005). Food restriction in general, or protein restriction specifically, during pregnancy, can reduce 11β-HSD2 in the placenta (Langley-Evans et al., 1996; Lesage et al., 2006). The foetus is therefore vulnerable to over-exposure to GC, which disrupts development of the HPA axis and leads to elevated hippocampal mineralocorticoid receptor expression and an exacerbated corticosterone response to stress in later life (Lesage et al., 2002, 2006). Some of these long-term effects of foetal exposure to GC can be prevented by exogenous inhibitors of GC synthesis (Langley-Evans et al., 1996).

### Postnatal nutrition

It is currently hypothesized that hyperphagia and obesity after *in utero* growth restriction are due to a mismatch between the developmental and subsequent environments leading to excessive “catch up growth” and associated changes in feeding behavior (Gluckman and Hanson, 2004; Wadhwa et al., 2009). Indeed, rapid weight gain in humans in the first week after birth is a highly significant risk factor for obesity in later life (Ong et al., 2000; Stettler et al., 2005). Remarkably, for every 100 g of weight gained in the first week of life, the risk of becoming obese as an adult increases by 28% (Stettler et al., 2005).

In rodents, as well as humans, the timing as well as the rate of catch up growth is important in determining its influence on development. If the *in utero* food restriction is continued after birth, by continuing to food-restrict the dam during lactation, the growth-restricted phenotype is exacerbated. That is, the animals remain small during the suckling period but by 9 months of age they are normal-weight, have normal levels of fat and leptin, and have improved glucose tolerance and insulin sensitivity (Desai et al., 2005; Berleze et al., 2009). Conversely, if the *in utero* food-restricted pups are suckled by an *ad-libitum*-fed dam, rapid catch up growth occurs and the pups become larger and fatter in adulthood than controls, and have elevated leptin levels (Desai et al., 2005). These rodent studies thus suggest preventing or delaying catch up growth removes the increased propensity to develop obesity (Desai et al., 2005; Ross and Desai, 2005). Supporting this are data from the 1941–1944 Siege of Leningrad famine. Because of the long duration of the famine and general food shortage and poor living standards before and after it (Leon et al., 1997; Roseboom et al., 2000a), it is likely those people subjected to *in utero* malnutrition continued to be fed a similar diet after birth and did not have significant catch up growth. In this cohort there was, consequently, no association between perinatal malnutrition and adult obesity or risk of disease (Stanner et al., 1997).

In rodents, a useful model for altered postnatal nutrition is to manipulate the litter sizes in which the pups are raised (McCance, 1962; Plagemann et al., 1999b; Schmidt et al., 2001; Morris et al., 2005; Plagemann, 2006; Chen et al., 2008; Rodel et al., 2008; Morris and Chen, 2009; Spencer and Tilbrook, 2009). Thus, rats and mice can be allocated to litters that are smaller than usual; usually three or four pups per litter compared with approximately 12 as a control. In this environment small-litter rodents drink more milk, and milk that is higher in fat and has a greater energy content than those from control litters (Fiorotto et al., 1991). Rodents that are suckled in small litters for the duration of the suckling period weigh significantly more in adulthood than those from control litters (McCance, 1962; Plagemann et al., 1999b; Schmidt et al., 2001; Morris et al., 2005; Plagemann, 2006; Chen et al., 2008; Rodel et al., 2008; Morris and Chen, 2009; Spencer and Tilbrook, 2009). They have more body fat in adulthood and have indices of metabolic disturbances (Plagemann et al., 1992). These small-litter rats also have pronounced changes in HPA axis function (Spencer and Tilbrook, 2009). Their HPA axes mature faster than in rats from control litters (Boullu-Ciocca et al., 2005) and they have exacerbated HPA axis responses to psychological (females) (Spencer and Tilbrook, 2009) and physical (females and males) stress (Clarke et al., 2012).

In conjunction with causing life-long weight gain and enhanced fat mass, many groups have seen small-litter rearing causes hyperphagia and increased appetite throughout life (Plagemann et al., 1999b; Biddinger and Fox, 2010). Mice raised in small litters eat more at each meal, have impaired satiety, and they eat their first meal faster following a mild food deprivation (Biddinger and Fox, 2010). This effect of postnatal overfeeding is likely to be due to changes in the hypothalamic circuitry that regulates feeding. It is interesting, however, that not all groups have seen hyperphagia in this model (Stefanidis and Spencer, 2012), again suggesting multiple influences are necessary to regulate feeding behavior.

The converse model to suckling rodents in small litters is to allocate them to large litters where their access to milk is reduced. Rats suckled in this manner have slower growth and remain smaller throughout life (Velkoska et al., 2008; Bulfin et al., 2011). They also have long-term changes in HPA axis function (Hernandez et al., 2010; Bulfin et al., 2011). Their HPA axis responses to stress (restraint) are attenuated, with corticosterone concentrations returning to baseline more quickly indicating the response may be more efficient in these animals (Bulfin et al., 2011).

Dietary composition is an important factor that can also potentially influence growth and development generally, and specifically development of the central circuitry that regulates feeding. The World Health Organization now recommends exclusive breast-feeding of infants for at least 6 months (WHO, 2002) and some investigations have illustrated this has a protective effect against obesity. For instance, exclusive breast-feeding has been linked with slower weight gain and a reduced long-term obesity risk compared with formula-feeding (e.g., Kramer et al., 2004; Holmes et al., 2011). There is some controversy to these findings and it may be that socio-economic status, related lifestyle factors, or the nutritional content of the formula are also important (Durmus et al., 2011). For instance, these differences in weight gain have not been shown in babies fed low-protein formula (Koletzko et al., 2009). In this regard, the content of the breast-milk also seems to be important (Rist et al., 2007). For instance conjugated linoleic acid isomers (found in organic dairy and meat products) can prevent fat deposition in some human trials (Racine et al., 2010) and these are increased in the breast-milk if the mother has a high dietary intake (Rist et al., 2007). Even the frequency of feeding may also affect the baby's risk of developing obesity (Erlanson-Albertsson and Zetterstrom, 2005; Toschke et al., 2005), as can the timing of the introduction of formula or solid food (Seach et al., 2010).

The perinatal period is thus extremely vulnerable to programming by nutritional influence and stress. Excess stress and/or inadequate nutrition during development can interact to influence feeding and stress responses long-term. Although the mechanism(s) by which these early life challenges influence this circuitry have not been definitively determined, we do have some indications as to how this occurs.

## Mechanisms by which the perinatal environment influences feeding and stress responses long term

The HPA axis is the principal neuroendocrine mechanism by which the body responds to a stressful event. When an animal perceives an actual (physical) or potential (psychological) threat (Dayas et al., 2001), medial parvocellular cells in the PVN at the apex of the HPA axis are activated. Corticotropin-releasing hormone (CRH) and arginine vasopressin (AVP) are then released into the median eminence, followed by ACTH release from the anterior pituitary into the blood stream. ACTH acts at melanocortin 2 receptors to stimulate GC release from the adrenal cortex. GC are the principal mediators of the stress response, encouraging, for example, memory formation, blood flow to skeletal muscle, and suppression of further HPA axis activity [reviewed in Sapolsky et al. (2000); Papadimitriou and Priftis (2009)].

**Figure 1:**
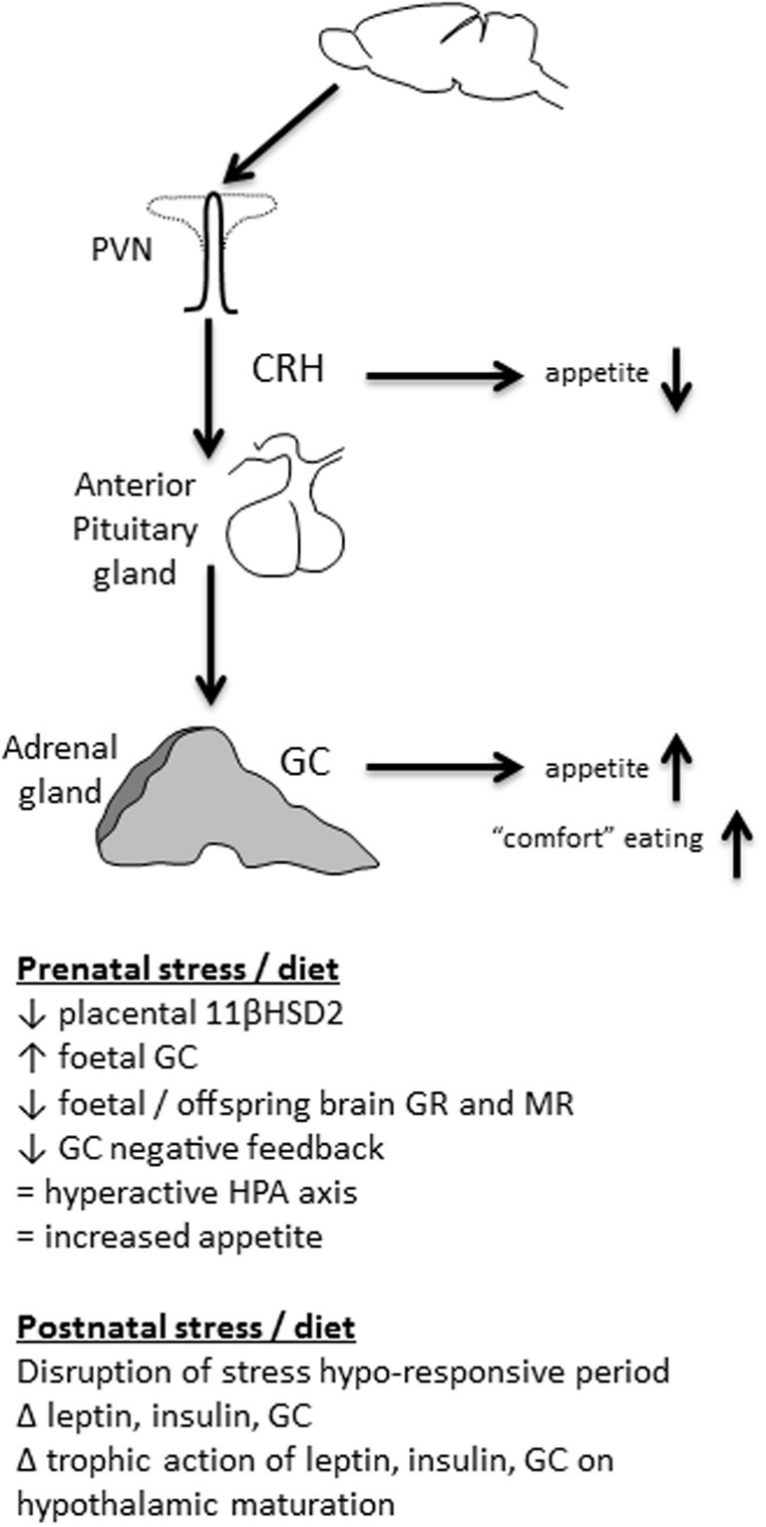
**The HPA axis can be programmed by early life stress and diet**. When an animal is stressed, medial parvocellular cells in the paraventricular nucleus of the hypothalamus (PVN) are activated. Corticotropin-releasing hormone (CRH) is released into the median eminence, followed by adrenocorticotropic hormone (ACTH) release from the anterior pituitary into the blood stream. ACTH stimulates glucocorticoid (GC) release from the adrenal cortex. Early in the stress response CRH mediates anorexia. As the stress response progresses, GC stimulate appetite, particularly for highly palatable food. Early life events that disrupt HPA axis function can therefore influence feeding behavior long-term.

The HPA axis generally, and GC in particular, are also crucial for appetite regulation. In the first minutes after a stressful event, appetite is typically suppressed; a mechanism that discourages food seeking and feeding when more pressing actions such as escape or defense are more prudent. This early stress-induced anorexia is mediated by CRH (Heinrichs and Richard, 1999; Richard et al., 2002), and other molecules of the same family, such as urocortins (Weninger et al., 1999; Richard et al., 2002). CRH influences food intake by acting at a number of appetite-regulatory brain regions including PVN, perifornical and ventromedial regions of the hypothalamus, lateral septum, and parabrachial nucleus (Richard et al., 2002), as well as indirectly influencing the dorsal anterior bed nucleus of the stria terminalis (Ciccocioppo et al., 2003). CRH and urocortins probably inhibit the activity of neuropeptide Y (NPY) neurons at the hypothalamus, which would normally stimulate food intake (Heinrichs et al., 1993; Currie, 2003). In this regard, any change in expression of CRH or its receptor is therefore likely to influence appetite (Figure 1). For instance, rats suckled in small litters, where they develop long-term hyperphagia and obesity have suppressed hypothalamic CRH expression, potentially contributing to less appetite suppression than in controls (Boullu-Ciocca et al., 2005).

Although CRH stimulated by stress acutely suppresses appetite, GC have the opposite effect in that they stimulate feeding (Santana et al., 1995; Dallman et al., 2004; Figure 1). GC are released downstream from CRH and can stimulate appetite for hours to days after a stressful event. In humans, a peripheral injection of CRH stimulates food intake 1 h later and this food consumption is directly related to the size of the peak cortisol response (George et al., 2010). After an acute stressor this encourages the animal to replace energy stores that were lost coping with the stress. If the HPA axis is hyperactive, however, with more GC secreted in response to stress, this can lead to a chronically stimulated appetite and increased feeding (De Vriendt et al., 2009).

Thus, one explanation for how perinatal stress and nutritional challenge can influence the neuroendocrine mechanisms regulating feeding behavior long-term is that they can permanently alter the sensitivity of the HPA axis and thus the levels of GC the animal is exposed to. As discussed, despite a number of mechanisms to protect the foetus, maternal GC can cross the placenta and influence foetal GC levels, receptor expression (Edwards et al., 1993), and 11β-HSD2 (Clifton et al., 2006). Foetal 11β-HSD2 levels are important in programming subsequent stress responses and 11β-HSD2^−/−^ mice have greater anxiety levels and lower birth weights than wildtype littermates (Holmes et al., 2006). Similarly, stress (e.g., maternal separation) in the postnatal period can lead to a disruption of the stress hypo-responsive period and elevated GC (Wigger and Neumann, 1999; Lajud et al., 2012).

Excess GC in the foetus or neonate have a number of effects. They interfere with synaptic pruning during brain development in regions that are important for the control of the HPA axis; the prefrontal cortex and hippocampus (Jacobson and Sapolsky, 1991; Spencer et al., 2005). Thus, when dams are given restraint stress during pregnancy, the offspring have reduced levels of growth-associated protein of 43 kDa (GAP-43), which is normally responsible for establishing appropriate synaptic connectivity during development (Pfenninger et al., 1991; Larsson, 2006; Jutapakdeegul et al., 2009). Excess foetal or postnatal GC can also lead to reduced expression of GRs in the hypothalamus and hippocampus resulting in a less efficient GC negative feedback (Liu et al., 1997). This effect is likely to be, at least in part, epigenetically mediated. For example, rats given little parental attention (low intensity nursing) have a smaller increase in nerve growth factor inducible factor A (NGFI-A) expression when they are groomed (Hellstrom et al., 2012). NGFI-A increases histone acetylation of the GR, which facilitates demethylation of the GR promoter and thus receptor activity. With low levels of maternal attention, this activity is reduced, and this is reflected in hypersensitive HPA axis responses to stress in these animals (Champagne and Meaney, 2001). The opposite occurs with high levels of maternal attention (Fish et al., 2004; Meaney and Szyf, 2005). Interestingly, these changes in HPA axis function due to differences in maternal care seem to be insufficient to alter feeding behavior under basal conditions, as no differences in adiposity or feeding have been reported in these studies (Connor et al., 2012). Epigenetic processes are likely to mediate many changes in HPA axis function as a result of early life events. For instance, in addition to the studies of Meaney and colleagues, early life maternal separation stress in mice leads to changes in DNA methylation leading to increased AVP expression in the PVN, elevated basal GC, and changes in stress-coping style (Murgatroyd et al., 2009; Murgatroyd and Spengler, 2011). There is also some evidence to suggest epigenetic modifications can influence the interaction between stress and feeding behavior. Thus, maternal undernutrition results in increased histone acetylation and hypomethylation of the GR in the offspring's hypothalamus, with a substantial increase in GR expression in this region. These changes are closely linked with enhanced weight gain in these offspring (Stevens et al., 2010; Begum et al., 2012).

**Figure 2:**
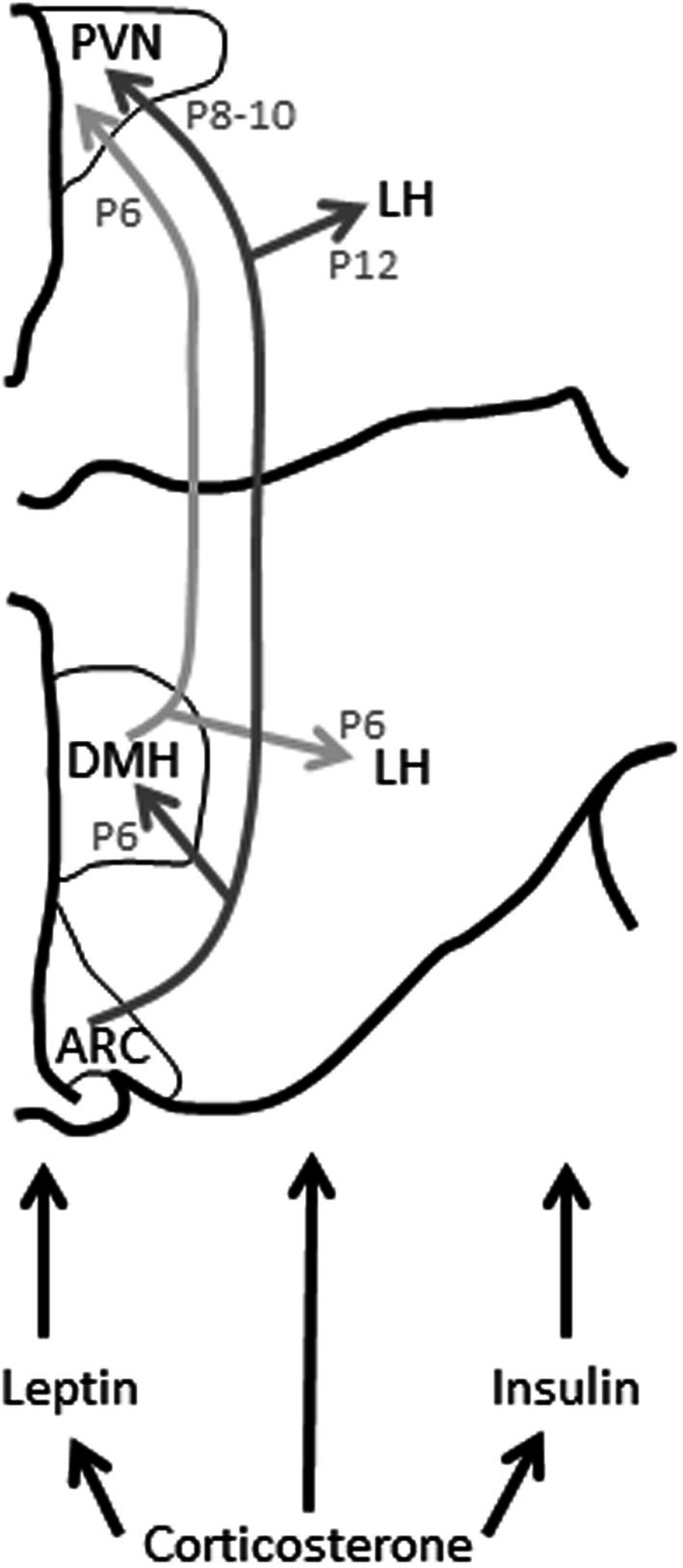
**Leptin, insulin, and glucocorticoids modulate hypothalamic connectivity during development**. Changes to these hormonal profiles in early life can influence the development of this connectivity and thus feeding behavior and, probably, HPA axis function long-term. ARC, arcuate nucleus of the hypothalamus; DMH, dorsomedial nucleus of the hypothalamus; LH, lateral hypothalamus; P, postnatal day (day by which this connectivity has developed in the rat); PVN, paraventricular nucleus of the hypothalamus. Figure adapted from Bouret and Simerly (2006).

During development and in adulthood, GC also modulate the activity of other hormones involved in regulating feeding, including leptin (Spencer, 2012). In adults, leptin, secreted from adipocytes acts centrally, particularly at the arcuate nucleus of the hypothalamus (ARC) to suppress appetite (Schwartz et al., 2000). Although GC stimulate leptin release from adipose tissue, which would normally lead to appetite suppression, they also reduce the sensitivity of the brain to leptin, contributing to leptin resistance (Zakrzewska et al., 1997, 1999; Jequier, 2002). Additionally, in the developing animal, leptin is an important trophic factor, stimulating the development of the brain pathways that regulate feeding (Vickers et al., 2005, 2008; Bouret and Simerly, 2007), and excess GC can interfere with this development (Figure 2).

From birth in rodents and from approximately gestational day 21 in humans, the hypothalamic pathways subserving feeding start to fully mature (Koutcherov et al., 2002; Bouret and Simerly, 2006). In rodents, connectivity between the ARC and the dorsomedial hypothalamus (DMH) are established by postnatal day (P) 6, the ARC and PVN by P8-P10, and the ARC and lateral hypothalamus (LH) by P12 (Bouret et al., 2004a,b). This connectivity is dependent upon a surge in circulating leptin that occurs between P4 and P16 (Ahima et al., 1998), the leptin acting on the brain to trigger these communication pathways to mature. If this leptin surge does not occur at the crucial time, this connectivity is improperly formed, permanently affecting feeding-regulation (Bouret and Simerly, 2007). Leptin-deficient mice (Lep^ob^/Lep^ob^) that do not have this surge have impaired connectivity between the ARC, DMH, PVN, and LH, with a significant decrease in axon density between these regions. They are also hyperphagic and develop obesity in later life. This connectivity can be rescued by daily supplementation with leptin between, but not after, P4 and P12 (Bouret et al., 2004a,b; Vickers et al., 2005, 2008).

The leptin surge and the connectivity of this hypothalamic circuitry can be disrupted by maternal and postnatal diet and by early life stress. For instance, *in utero* growth restriction can lead to a premature leptin surge and a disruption of these hypothalamic pathways, an effect that is mimicked by exogenous leptin administration from P5 to P10 (Yura et al., 2005). Similarly, rats born to obese dams have elevated plasma leptin in the first 3 weeks of life, and this is associated with resistance to the anorexigenic effects of leptin, hyperphagia, and obesity long-term (Kirk et al., 2009). Postnatal rodents receive much of their leptin from their mother's milk (Fiorotto et al., 1991). Therefore, pups raised by hyperleptinemic mothers, or in small litters where they drink more milk, may be exposed to more leptin than is optimal. It is likely this excess leptin triggers a premature leptin surge, thus reprogramming these feeding-regulatory pathways. Additionally, postnatal overfeeding, or even a genetic susceptibility to diet-induced obesity, can lead to an insensitivity of the ARC to leptin (Davidowa and Plagemann, 2000; Bouret et al., 2008). For example, ARC explants from diet induced obesity-susceptible rats are less responsive to leptin, developing less neurite outgrowth after leptin application than controls (Bouret et al., 2008). In humans, the equivalent period of development is likely the third trimester of gestation. In humans, intrauterine growth restricted babies or those born small for gestational age have reduced circulating leptin compared with normal babies (Ren and Shen, 2010). Conversely, babies born to obese mothers may be exposed to high levels of leptin too early (Catalano et al., 2009). In either case, if the timing or magnitude of the leptin surge is disrupted, the development of the necessary hypothalamic circuitry may not take place appropriately and feeding behavior would be permanently altered.

As well as an effect of early diet on leptin, it is clear excess GC during the perinatal period can influence the leptin-dependent development of this circuitry. Administration of the synthetic GC, dexamethasone, from postnatal day (P)3 to P6 can elevate plasma leptin in rats, which may interfere with the leptin surge (Bruder et al., 2005). In humans, betamethasone given to pregnant women significantly enhances their plasma leptin concentrations and also elevates leptin levels in the foetus and neonate (Marinoni et al., 2008). Conversely, elevated leptin during the neonatal period can alter HPA axis function long-term, increasing GR levels in the PVN and hippocampus, and enhancing the efficiency of the GC negative feedback response to stress (Proulx et al., 2001).

In addition to modulating the effects of leptin, GC also contribute to insulin secretion from the pancreas and can modify insulin's action on the brain (Strack et al., 1995). Insulin usually acts to suppress appetite via its actions at the hypothalamus and suppresses dopamine-mediated reward at the ventral tegmental area (Figlewicz et al., 2008). However, insulin also enhances preference for high fat, high sucrose “comfort foods” (La Fleur et al., 2004; Warne et al., 2006, 2009). So, chronically elevated GC or a hyperactive GC response to stress programmed by early life events can contribute to comfort eating. Elevated GC also reduce insulin's ability to inhibit feeding-stimulatory pathways in the brain, again leading to inappropriate feeding behavior (Asensio et al., 2004).

Insulin signaling in the adult is sensitive to the early life developmental environment and maternal over-nutrition leads to long-term changes in insulin secretion and sensitivity (Plagemann, 2008). As with leptin, insulin also acts as a trophic factor during development to stimulate connectivity of appetite-regulating brain pathways (Figure 2). For instance, excess insulin prenatally, due to hyperinsulinemia in the mother, or postnatally due to overfeeding, can lead to higher insulin concentrations at the level of the hypothalamus, and this may result in changes in brain cell morphology (Plagemann et al., 1998). In particular, the neurons of the ventromedial hypothalamus are vulnerable to insulin, and excess central insulin can lead to a permanent increase in orexigenic NPY and galanin in the ARC and PVN at weaning and in later life (Plagemann et al., 1992, 1999a,b).

It is not clear how, or if, perinatal stress can influence the programming effects of insulin on the circuitry regulating feeding. As with leptin, perinatal stress can stimulate an increase in circulating insulin (Moyer-Mileur et al., 2011). Perinatal stress also clearly affects adult HPA axis function and this can significantly alter how the brain responds to insulin. Future experiments are necessary to determine if acute changes in insulin as the result of stress in early life are sufficient to alter insulin-dependent brain development, or the effects of early life stress are less direct.

In addition to leptin and insulin, early life stress can influence feeding behavior long-term by influencing sensitivity to serotonin. For instance, *in utero* protein restriction combined with *ad-libitum* feeding postnatally can lead to a reduction in the anorexic effects of serotonin (Lopes De Souza et al., 2008). Serotonin sensitivity is also closely regulated by stress (Asan et al., 2013). GC may also further influence appetite regulation via their effects on ghrelin. Ghrelin's principal function is to stimulate feeding, but levels of this hormone are increased during stress and can modulate responses to several stressors (Hosoda et al., 2006; Kristenssson et al., 2006; Spencer et al., 2012). Conceivably, changes in HPA axis function and GC production as a result of the early life environment would alter ghrelin's ability to stimulate feeding. Details of the roles of serotonin and ghrelin in integrating stress and feeding behavior remain to be investigated.

Encouragingly, there is significant potential for effects of perinatal programming on endocrine mechanisms connecting feeding behavior and stress to be reversed or ameliorated by concomitant or later dietary or other interventions. For instance, a postnatal diet high in omega-3 fatty acids ameliorates hyperleptinemia and hypertension associated with *in utero* exposure to dexamethasone (Wyrwoll et al., 2006). Interventions to normalize leptin have also been particularly successful in animal models. Thus, the reduction in 11β-HSD2 associated with a maternal low protein diet during pregnancy can be normalized by giving the dams leptin throughout pregnancy and lactation (Stocker et al., 2004). Neonatal treatment with leptin, from P3 to P13, can also reduce the hyperleptinemia, hyperinsulinemia, hyperphagia, and obesity associated with *in utero* growth restriction and subsequent high fat diet, albeit with some sex differences (Vickers et al., 2005, 2008). Such treatments can even be effective into adulthood, with insulin-like growth factor-1 treatment in adult rats also reducing the long-term effects of *in utero* growth restriction on feeding and metabolic sequelae (Vickers et al., 2001).

In summary, the early life environment has a critical role in programming the circuitry that later integrates stress and feeding behavior. A hyperactive HPA axis, programmed as the result of a stressful early life environment, can lead to excess GC and an exacerbation of GC's typical appetite-stimulatory effects. GC's interactions with feeding-related hormones such as leptin and insulin are also affected. In addition, the early life environment also has specific influences on brain development, such as ensuring appropriate connectivity between the various parts of the hypothalamus necessary for regulating feeding. Stress and the early life nutritional environment can acutely affect this brain development leading to abnormal feeding behavior long-term.

## Acknowledgments and funding sources

This work was supported by a Discovery Project Grant from the Australian Research Council (ARC) to Sarah J. Spencer (DP130100508), and Project Grant from the National Health and Medical Research Council (NHMRC) to Dr Zane Andrews and Sarah J. Spencer (APP1011274). Sarah J. Spencer is an ARC Future Fellow (FT110100084) and an RMIT University VC Senior Research Fellow.

### Conflict of interest statement

The author declares that the research was conducted in the absence of any commercial or financial relationships that could be construed as a potential conflict of interest.
